# Coxsackievirus-B4 Infection Can Induce the Expression of Human Endogenous Retrovirus W in Primary Cells

**DOI:** 10.3390/microorganisms8091335

**Published:** 2020-09-01

**Authors:** Arthur Dechaumes, Antoine Bertin, Famara Sane, Sandrine Levet, Jennifer Varghese, Benjamin Charvet, Valéry Gmyr, Julie Kerr-Conte, Justine Pierquin, Govindakarnavar Arunkumar, François Pattou, Hervé Perron, Didier Hober

**Affiliations:** 1 Laboratoire de Virologie ULR3610 Univ Lille, CHU Lille, 59000 Lille, France; a.dechaumes@gmail.com (A.D.); antoinebertinb@gmail.com (A.B.); famara.sane@chru-lille.fr (F.S.); jennifervarghese5@gmail.com (J.V.); 2 Geneuro Innovation, 69008 Lyon, France; sl@geneuro.com (S.L.); benjamin.charvet@geneuro.com (B.C.); jp@geneuro.com (J.P.); hperron@geneuro.com (H.P.); 3 Manipal Institute of Virology, Manipal Academy of Higher Education, Karnataka 576104, India; arunviro@gmail.com; 4 U1190 Univ Lille, Inserm, CHU Lille, European Genomic Institute for Diabetes, 59000 Lille, France; vgmyr@univ-lille.fr (V.G.); jkerr-conte@univ-lille.fr (J.K.-C.); francois.pattou@univ-lille.fr (F.P.); 5 Geneuro SA, 1228 Geneva, Switzerland; 6 Faculté de Médecine Laënnec, Université de Lyon, 69008 Lyon, France

**Keywords:** enterovirus, endogenous retrovirus, pancreatic cells, macrophages, PBMCs

## Abstract

Human Endogenous Retrovirus W Envelope (HERV-W ENV) mRNA or protein can be found in peripheral blood mononuclear cells (PBMCs) and exocrine pancreas of patients with type 1 diabetes (T1D). Further, previous observations have shown an association between enteroviral infection and development of T1D; specifically, coxsackievirus-B (CV-B) has been detected in the blood and pancreas of patients with T1D. Notably, viruses can activate HERV-W expression. Hence, we evaluated the effect of CV-B4 infection on HERV-W *ENV* mRNA expression. Primary human pancreatic ductal cells were obtained from five brain-dead donors. In the pancreatic cells of three donors, the HERV-W *ENV* mRNA level measured using RT-qPCR was upregulated upon CV-B4 infection. The HERV-W ENV protein was detected in the infected cells using the immunoblot assay. In human PBMCs inoculated with CV-B4 or when CV-B4 was incubated with an enhancing serum, the HERV-W *ENV* mRNA level was higher than the background RNA level. In monocyte-derived macrophages obtained from 5 of 13 donors, the HERV-W *ENV* mRNA level was higher in cultures inoculated with CV-B4 than in the control. Therefore, CV-B4 can upregulate or induce the transcription of a certain HERV-W *ENV* copy (or copies) in primary cell cultures, such as monocytes, macrophages, and pancreatic cells.

## 1. Introduction

Human Endogenous Retroviruses (HERVs) are vestigial sequences endogenized in primate germinal cells and transmitted across generations over the course of evolution [1]. HERVs are disseminated in the genome through retrotransposition and recombination, resulting in creating multiple copies of HERV genes [2]. HERVs account for approximately 8% of the human genome, but most copies are silenced by mutations, deletions, or epigenetic modifications [3]. Some belong to the pool of physiological genes; Syncytin-1, which is an envelope protein of HERV-W, is involved in placentation [4].

There are extensive interindividual variations in the proviral content of HERV families in human genomes [5,6]. In contrast with domesticated HERV copies, which have physiological roles like Syncytin-1, HERVs may be involved in the pathogenesis of some diseases, such as autoimmune diseases, relying on a complex interplay between environmental and genetic factors. HERVs may assist in drawing a link between environmental factors, genetic factors, and pathogenic mechanisms of diseases such as multiple sclerosis (MS) or amyotrophic lateral sclerosis [7]. The envelope protein of HERV-W, termed HERV-W ENV, is particularly being studied in autoimmune diseases, given its immunopathogenic properties [8]. Previous studies have revealed an association between HERV-W ENV and type 1 diabetes (T1D) supported by the detection of HERV-W ENV using various methods (enzyme-linked immunosorbent assay (ELISA), RT-qPCR, or immunohistochemistry (IHC)) in human serum, peripheral blood mononuclear cells (PBMC), or pancreas. In patients with T1D, HERV-W ENV was detected widespread or in clusters in the exocrine pancreas and in the vicinity of Langerhans islets [3].

Viruses belonging to the Herpesviridae family can trigger the expression of HERVs involved in the development of MS [9,10,11]. Further, epidemiological and experimental studies support the hypothesis of an involvement of enteroviruses, notably coxsackieviruses B (CV-B), such as CV-B4, in the development of T1D [12,13,14]. CV-B4, like the five other serotypes of CV-B (CV-B1, B2, B3, B5, and B6), belongs to Human Enterovirus B species present in the Enterovirus genus of the Picornaviridae family [15]. Enteroviruses are small (approximately 30 nm), non-enveloped, positive-sense single-stranded RNA viruses (approximately 7500 nucleotides) with an icosahedral capsid symmetry. The capsid is composed of 60 capsomers encompassing a single copy of each of the structural proteins VP1, VP2, VP3, and VP4. CV-B, like all the members of the Enterovirus genus, replicates in the cytoplasm of the cell [16]. The first step involves attachment to a cellular receptor. CV-B4 requires the coxsackievirus adenovirus receptor (CAR) for entry and uncoating in host cells [17]. Markers of enteroviral infection, especially CV-B, have been found using molecular and immunological diagnostic techniques in various biological samples, especially the blood and pancreas of patients with T1D [12,18,19]. Enteroviral RNA was detected in ductal cells of the pancreas of patients with T1D [20]. Human pancreatic ductal cells can be persistently infected with CV-B4 in vitro, thus impairing the differentiation of these precursors in endocrine cells and disrupting the microRNA expression profile [21,22]. Further, the infection of monocytes with CV-B4 can be enhanced with non-neutralizing serum samples or IgGs targeting the VP4 capsid protein. The interaction between CV-B4, enhancing antibodies, and monocytes depends on the CV-B4 receptor CAR and FcγRII and FcγRIII receptors of the Fc portion of IgG [23,24,25]. Enteroviral RNA has been detected in monocytes of patients with T1D as compared with healthy controls [26], and human monocyte-derived macrophages are readily infected with CV-B4 in vitro [27]. Moreover, in vivo, monocytes and macrophages can be infected with CV-B4 [28].

In light of recent discoveries on the activation of HERV-W ENV in PBMCs and pancreatic cells of patients with T1D [3] and considering the association between CV-B, especially CV-B4, and T1D, we aimed to evaluate the potential effect of CV-B4 on the expression of HERV-W *ENV* in these cells.

## 2. Materials and Methods

### 2.1. Virus

The diabetogenic strain CV-B4 E2 was provided by Ji-Won Yoon (Julia McFarlane Diabetes Research Center, Calgary, AB, Canada), and the CV-B4 JVB strain was provided by J Almond (Aventis Pasteur, Marcy l’étoile, France). Both strains were propagated in HEp-2 cells (BioWhittaker, Walkersville, MD, USA).

### 2.2. Human Serum

Human serum sample with anti-CV-B4 enhancing activity was selected as previously described by our team [25,27]. Briefly, when peripheral blood mononuclear cells (PBMCs) cultures are inoculated with CV-B4 mixed with diluted human immune serum, the level of intracellular enteroviral RNA is higher than the one in PBMCs cultures inoculated with CV-B4 [25,27].

### 2.3. Human Pancreatic Cells

Human pancreatic cells were harvested from brain-dead adults in agreement with the French law and ethical committee of our institution. The exocrine fraction was extracted and processed as described previously [29,30,31]. Briefly, pancreatic cells were seeded in six-well plates in Dulbecco’s Modified Eagle Medium (DMEM) containing 3 g/L glucose, 10% fetal bovine serum (FBS) (Gibco, Thermofisher Scientific, Courtaboeuf, France), 1% insulin transferrin selenium (Sigma-Aldrich, Saint-Quentin-Fallavier, France), and 1% penicillin/streptomycin (Gibco), as well as 50 µg/mL Geneticin (G418, Sigma-Aldrich) to limit fibroblast overgrowth. The cells were incubated at 37 °C supplemented with 5% CO_2_, and a monolayer was obtained 48–96 h later. Cells were then inoculated with 10^4^ to 10^6^ TCID50/mL of either CV-B4 E2 or CV-B4 JVB.

### 2.4. Peripheral Blood Mononuclear Cells

Whole blood samples obtained from donors were subjected to density gradient centrifugation using the Ficoll-Hypaque TM PLUS medium (GE Healthcare, Vélizy-Villacoublay, France) at 400G/20 °C for 40 min. Peripheral blood mononuclear cells were isolated from the buffy coat layer and resuspended in non-supplemented Roswell Park Memorial Institute medium 1640 (RPMI, Gibco). The cells were rinsed twice in RPMI 1640 medium at 400 G/20 °C for 10 min. Thereafter, cells were seeded at an average of 5 × 10^6^ cells/well (~5 million/cm^2^) in Falcon^®^ polystyrene 1.5 mL tubes (Thermo Fischer Scientific, Illkirch-Graffenstaden, France). Non-supplemented RPMI 1640 medium completed with 10% FBS, 1% glutamine, and 1% streptomycin-penicillin was used. CV-B4 E2 at a multiplicity of infection (MOI) of 1 was pre-incubated with human serum (1:1000 and 1:10,000 dilution) at 37 °C for 2 h, 5% CO_2_, and inoculated into PBMCs. Cell pellets were rinsed six times using centrifugation at 400G/20 °C for 5 min in 1× Dulbecco’s phosphate-buffered saline (Gibco) and resuspended in completed RPMI 1640 medium after 4 h. Monocytes were then enriched by adherence to the plate incubated overnight and washed once to remove non-adherent cells and passively attached or unattached viral particles, with further incubation at 37 °C for 24 h, 5% CO_2_.

### 2.5. Human Macrophages

PBMCs were cultivated in six-well plates with 10^7^ cells/well. After an incubation period of 16 h, monocytes attached to the plastic surface, while other cells floated. Monocytes were then washed twice with RPMI 1640 medium, cultivated using Macrophage Serum Free medium (Gibco) with 20 µg/mL of macrophage colony-stimulating factor (M-CSF, PreproTech, Neuilly-sur-Seine, France), and incubated at 37 °C, 5% CO_2_ for 7 days. At the end of the incubation, monocytes were differentiated into macrophages and infected with CV-B4 [27].

### 2.6. Viability Measurement

Orangu^TM^ (Cell Guidance Systems, Cambridge, UK) was used to measure cell viability according to the manufacturer’s instructions. Cells were incubated for 6 h at 37 °C, 5% CO_2_, with 10% Orangu^TM^. Supernatants were harvested, and optical density at 450 nm was read with Multiskan GO (Thermofisher Scientific). In all experiments aimed to evaluate the expression of HERV-W, the layers were washed to eliminate unattached cells. Thus attached living cells were selected. The marker was evaluated compared to beta-actin and hence compared to living cells.

### 2.7. Viral Titration

Culture supernatants were harvested and centrifuged at 400 G for 10 min. The pellet was discarded, and supernatants were stored at −80 °C before titration. HEp-2 cells were plated at 10^4^ cells/well in 96-well plates and incubated for 24 h. Supernatants were diluted 10-fold up to 10^−10^, and 100 µL of each dilution was inoculated into the HEp-2 cells. Plates were incubated for 48 h, and the cytopathic effects were visualized under an inverted microscope. Viral titers were determined with the Spearman–Karber formula and expressed as TCID50/mL.

### 2.8. RNA Extraction

RNA was extracted from the cells using the TriReagent^®^ RNA isolation reagent/chloroform procedure (Sigma-Aldrich). Extracted RNA was dissolved in 30 μL of diethylpyrocarbonate (DEPC)-treated water (Sigma-Aldrich). The RNA yield was quantified using μdrop on Multiskan GO Spectrophotometer (Thermo Fisher Scientific), and yield ≥ 50 ng/μL was used in RT-qPCR assays. An enzymatic digestion of DNA products was performed on the extracted RNA samples. A volume of 10 μL of the RNA sample was mixed with 2 μL of DNase RDD 10× buffer (QIAGEN, Hilden, Germany), 0.2 μL of DNase I (Qiagen), 0.25 μL of RNase inhibitor (Roche Diagnostics, Meylan, France), and 7.55 μL of DEPC-treated water (Sigma-Aldrich). The incubation conditions were 37 °C for 30 min, 65 °C for 5 min, and hold at 4 °C.

### 2.9. Quantitative RT-PCR

#### 2.9.1. HERV-W *ENV* mRNA

RNA from cell cultures was retrotranscribed with cDNA synthesis kit (Invitrogen) before quantitative PCR was performed using iQ SYBR Green Supermix kit (Biorad) on Mx3000p^®^ (Stratagene/Thermo Fischer Scientific). The oligonucleotide primers (Eurofins Genomics, Les Ulis, France) are listed in Table 1. Primers were used at a final concentration of 50 nM. Cycling conditions were as follows: initial denaturation, 95 °C, for 5 min; denaturation, 95 °C, for 10 s; annealing, 60 °C, for 30 s; and extension, 72 °C, for 30 s, for a total of 40 cycles. The expression of HERV-W *ENV* mRNA was calculated after normalization with beta-actin gene, which was used as an endogenous control (housekeeping gene). The relative expression of HERV-W *ENV* mRNA was calculated in comparison with its expression in uninfected cells by using the 2^−ΔΔCt^ method [32]. CV-B4 infection is considered to have an effect on the expression of marker compared with mock control when the relative expression is higher than 2.

The oligonucleotide primers and probe (Eurofins Genomics) are listed in Table 1. HERV-W primers sequences were designed from cloned cDNA encompassing env-LTR sequence from virus-like particle released in MS culture supernatants (GenBank: AAK18189.1) [3]. The extent of variations in copies of HERV-W is limited. The primers target a region without significant variation as previously reported [3]. They can amplify the known HERV-W ENV copies.

#### 2.9.2. Enteroviral RNA

The synthesis of cDNA and its amplification was performed in a single tube using the TaqMan^®^ Fast Virus 1-Step Master Mix 4× (Applied Biosystems, Thermo Fischer Scientific) according to the manufacturer’s instructions. The oligonucleotide primers and probe (Eurofins Genomics) are listed in Table 2. The expression of Enteroviral RNA was calculated after normalization with beta-actin gene, which was used as an endogenous control (housekeeping gene).

### 2.10. Protein Extraction

Cell pellets were lysed in RIPA extraction buffer (Sigma-Aldrich) with 1% Fos-Choline-16 (Anatrace, Maumee, OH, USA) and protease inhibitors (Roche). Samples were incubated for 2 h at 25 °C under agitation. The total protein was measured with Protein Assay Reagent (Pierce, Thermofisher).

### 2.11. Quantification of HERV-W ENV Protein

The HERV-W ENV protein was measured using automated Western blot WES (ProteinSimple, Noyal Châtillon sur Seiche, France). Tests were applied according to the manufacturer’s instructions; 4 µL of the sample was mixed with the “fluorescent master mix” and incubated at 95 °C for 5 min. The blocking buffer, washing buffer, murine antibody GN_mAb_Env01 (Geneuro), horseradish peroxidase-conjugated secondary antibody (ProteinSimple), Mouse Detection Module, and chemoluminescent substrate were all loaded into their appropriate slots in the microplate. Separation and immunodectection were then automatically performed in capillary tracts using the default parameters [33].

## 3. Results

### 3.1. CVB4 Can Promote the Expression of HERV-W ENV in Primary Pancreatic Ductal Cells

**Figure 1:**
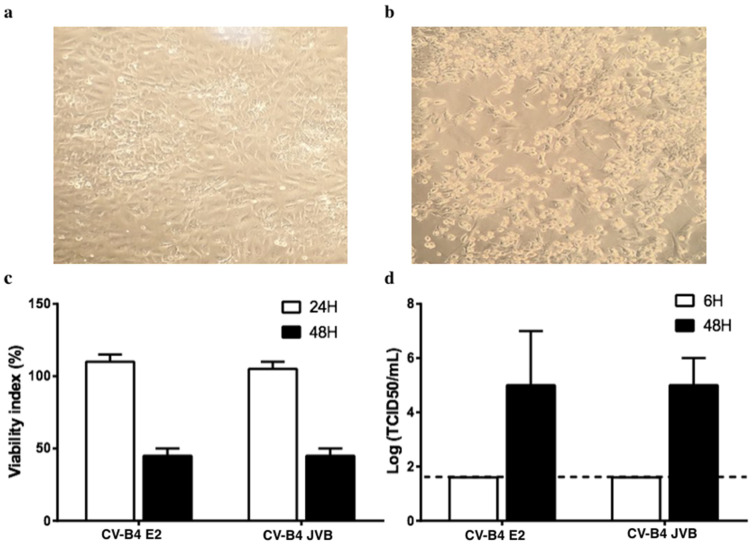
Cultures of primary human pancreatic ductal cells infected with Coxsackievirus B4 (CV-B4). (**a**) Pancreatic primary cells were obtained from five brain-dead donors. (**b**) Cultured cells infected with CV-B4 E2 or CV-B4 JVB (multiplicity of infection (MOI): 0.1) and observed under an inverted microscope (magnification ×100) after 48 h of incubation. (**c**) Viability of the cultured cells assessed using Orangu^®^. Optical density values are normalized using the viability value of uninfected cells (mock = 100%). (**d**) The level of infectious viral particles in cultured supernatants harvested 6 and 48 h post-inoculation at MOI 0.1 determined by titration on HEp-2 cells. Results are expressed as TCID50/mL. Means ± SD of log-transformed values of five independent experiments are shown.

Pancreatic ductal cells were isolated from the pancreas of five brain-dead donors. These cells were maintained in culture, and when the cultures were confluent, they were inoculated with CV-B4; 48 h post-inoculation, a cytopathic effect involving approximately 50% of the cells was observed, which agreed with the pattern of viability as assessed using the Orangu^®^ viability assay (Figure 1a–c). The level of infectious particles released in the supernatants is represented in Figure 1d.

**Figure 2:**
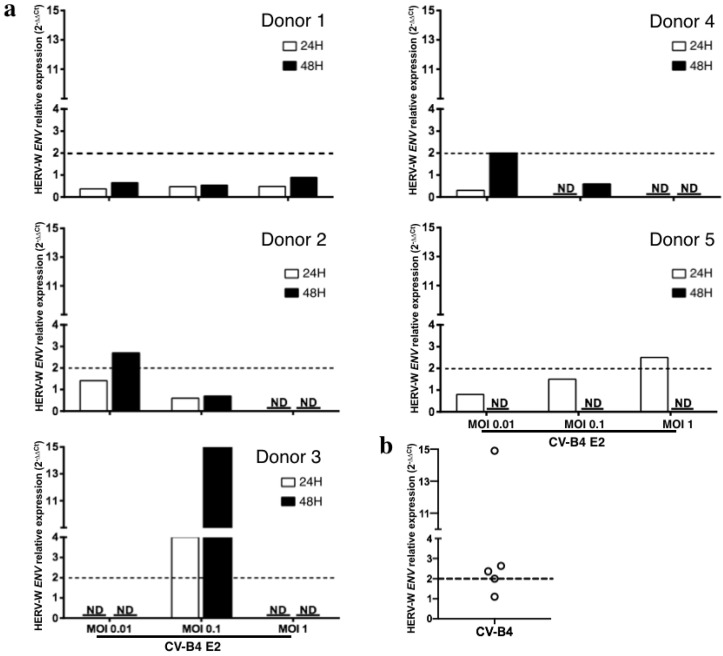
Human Endogenous Retrovirus W Envelope (HERV-W *ENV*) mRNA expression in primary human pancreatic ductal cells infected by Coxsackievirus B4 (CV-B4). Pancreatic primary cells obtained from five brain-dead donors were infected with CV-B4 E2 (multiplicity of infection (MOI) 0.01, 0.1 or 1). Total RNA was extracted from the cells with TriReagent, and the level of intracellular HERV-W *ENV* mRNA in each culture was assessed using RT-qPCR normalized with the level of beta-actin mRNA.Each result is the mean of two or three determinations; furthermore, each RT-PCR datum is the mean of two technical duplicates. The relative expression of HERV-W *ENV* mRNA in CV-B4 E2 infected cultures compared with that in mock-infected cultures was calculated using the 2^−ΔΔCt^ method. (**a**) The individual relative expression of HERV-W *ENV* for each culture is shown. ND: not done due to an insufficient number of cells. (**b**) The maximum individual relative expression of HERV-W *ENV* for each culture is shown. The dotted line indicates a relative expression of 2.

The transcriptional level of HERV-W *ENV* in cultured pancreatic ductal cells was quantified using RT-qPCR. HERV-W *ENV* expression was normalized with the housekeeping gene beta-actin. The relative expression of HERV-W *ENV* mRNA in CV-B4-infected cultures was > 2-fold higher than that in mock-infected cultures from three of five donors, peaking between 24 and 48 h post-infection in cultures infected at MOI 0.01, 0.1, and 1. In cells from donors 2, 3, 4, and 5, the peak relative expression was 2, 2.63, 2.36, and 14.9 respectively, whereas in cells from donor 1, the highest value was 0.89 (Figure 2a). As experimentation with primary cultures implicitly results in asynchronous infections and variable kinetics of HERV-W transcriptional induction between cells and cultures, we chose to compare peaks of mRNA quantification to address the issue of HERV-W transcriptional activation by CV-B4 (Figure 2b). This is globally indicating a transactivation by CV-B4 infection that is anyhow not expected to be synchronized between individuals if occurring in vivo with various enteroviral infectious doses reaching pancreatic cells.

**Figure 3:**
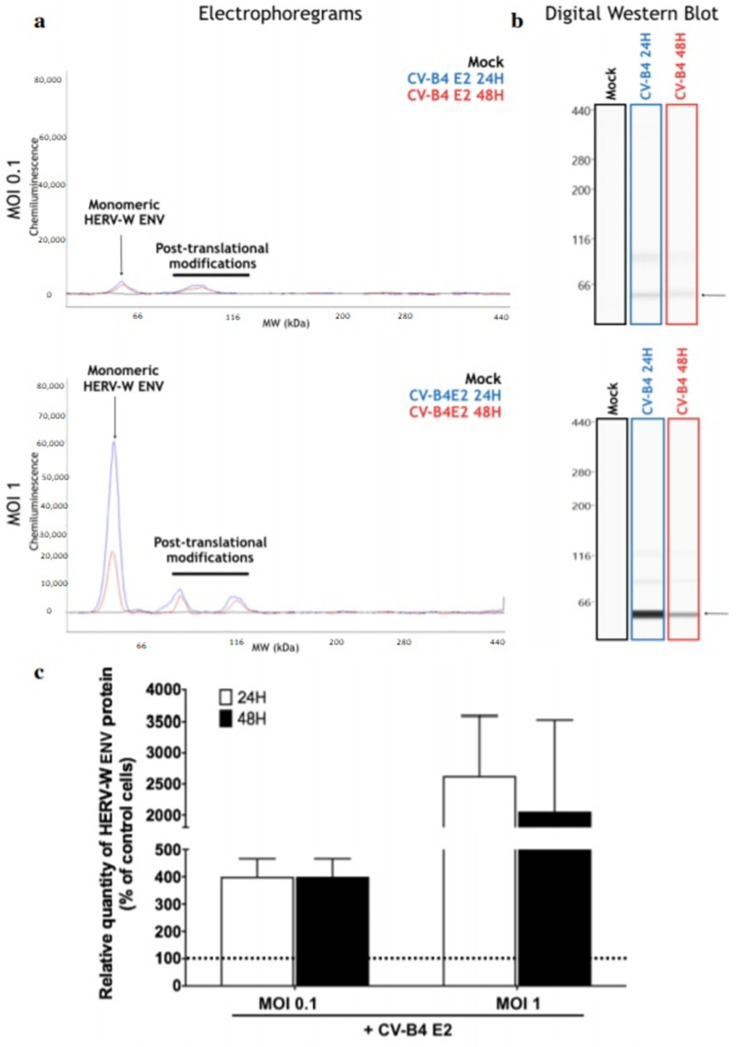
Levels of Human Endogenous Retrovirus W Envelope (HERV-W ENV) protein in primary pancreatic ductal cells infected by Coxsackievirus B4 (CV-B4). The levels of intracellular HERV-ENV protein in ductal cells obtained from two brain-dead donors were evaluated using capillary automated Western blot (Simple Western technology, ProteinSimple^®^). The cultures were inoculated with CV-B4 E2 at multiplicity of infection 0.1 and 1. Total protein lysates were analyzed on a 66-440kDa separation matrix, and HERV-W Env was detected using GN_mAb_Env01 antibody. (**a**) Electrophoregrams provided the migration profile of the target, and the intensity of the specific signal was precisely quantified using the area under curve (AUC) determination. (**b**) Signal quantification of the main peak corresponding to the monomeric form of HERV-W Env not carrying post-translational modifications (black arrow) was presented in a digital Western blot. (**c**) Results are expressed as the relative quantity of ENV in infected cultures compared with the non-specific background signal in mock-infected cultures. Results are presented as the mean ± SD of two independent experiments. The dotted line indicates the non-specific background signal of mock-infected cultures.

The level of HERV-W ENV protein in primary ductal cells infected by CV-B4 E2 was evaluated using automated Western blot. When cell cultures were inoculated with CV-B4 E2 (MOI 0.1), the levels of HERV-W ENV protein at 24 and 48 h post-infection were four times greater than the non-specific background signal of the corresponding mock-infected cultures. When cell cultures were inoculated with CV-B4 E2 (MOI 1), the levels of HERV-W ENV protein at 24 and 48 h post-infection were 25 times and 20 times greater, respectively than the threshold of mock-infected cultures (Figure 3).

### 3.2. CVB4 Can Promote the Expression of HERV-W ENV mRNA in PBMCs and Macrophages

#### 3.2.1. Infection of PBMCs

**Figure 4:**
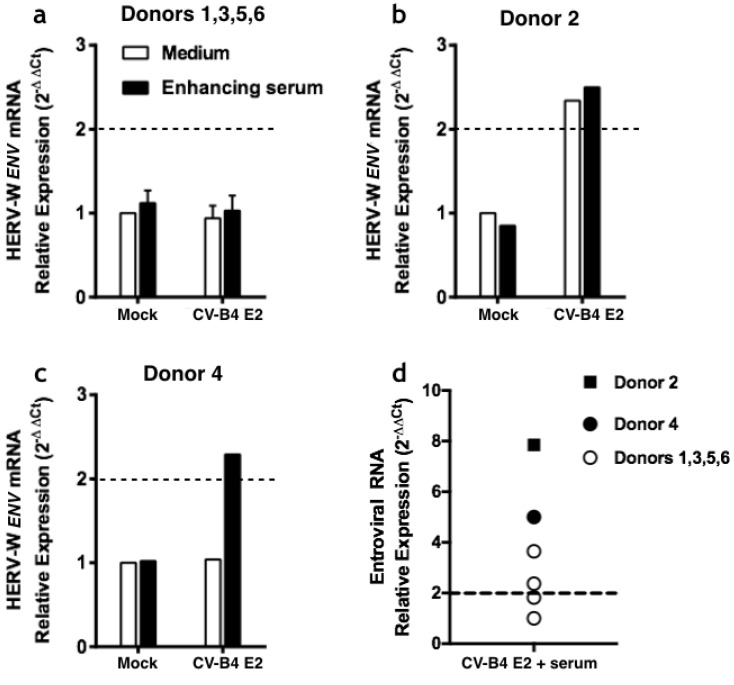
Relative expression of Human Endogenous Retrovirus W Envelope (HERV-W *ENV*) and enteroviral RNA in peripheral blood mononuclear cells (PBMCs) after inoculation with coxsackievirus B4 (CV-B4). PBMCs were isolated from buffy coats of six healthy donors and inoculated with CV-B4 E2 at multiplicity of infection 1 or with CV-B4 E2 mixed with human immune serum (dilution 1/1000), followed by incubation for 4 h. Thereafter, the cell layers were rinsed three times and incubated overnight. The cell layers were then washed once to eliminate non-adherent cells, and adherent cells were recovered to determine the level of HERV-W *ENV* mRNA and enteroviral RNA using RT-qPCR after normalization with beta-actin mRNA expression. For each condition, the result, which is the mean of two determinations, is the relative expression of the marker compared with the control calculated using the 2^–∆∆Ct^ method. Relative expression of HERV-W *ENV* mRNA in PBMCs from (**a**) donors 1, 3, 5, and 6 (mean ± SD), (**b**) donor 2, and (**c**) donor 4. (**d**) Individual representation of relative expression of enteroviral RNA in PBMCs of donors 1–6.

PBMCs were obtained from buffy coats of six healthy donors (Figure 4). The cells were cultured under various conditions followed by the recovery of adherent cells. The level of HERV-W *ENV* in adherent cells was evaluated using RT-qPCR. Compared with the HERV-W *ENV* global level in control conditions, there was no increased expression when PBMCs from donors 1, 3, 5, and 6 were inoculated with CV-B4 E2 (MOI = 1) alone (Figure 4a), whereas an increased expression of HERV-W *ENV* was found in PBMCs from donor 2 (relative expression 2.34-fold greater than the mock culture) (Figure 4b). Human immune serum (dilution 1/1000) mixed with CV-B4 E2 enhanced the infection of PBMCs from donors 2, 3, and 4 as shown by the level of enteroviral RNA in these cells compared with that in the controls (relative expression 7.85-, 3.65-, and 5.02-fold greater than mock cultures, respectively) as well as enhanced the infection of PBMC from two other donors but to a lower extent (Figure 4d). In parallel, CV-B4 mixed with diluted immune serum (1/1000 dilution) increased or induced transcriptional upregulation of HERV-W *ENV* in donors 2 and 4 (relative expression 2.5- and 2.29-fold greater than mock cultures, respectively) (Figure 4a–c). Of note, CV-B4 infection of PBMCs from another donor was not enhanced by the immune serum (Figure 4d).

#### 3.2.2. Infection of Macrophages

**Figure 5:**
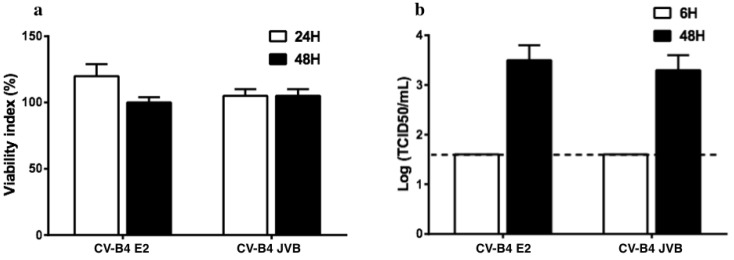
Infection of human primary macrophage cultures by Coxsackievirus B4 (CV-B4). Peripheral blood mononuclear cells from 13 healthy donors were isolated as previously described and cultured for 24 h. Thereafter, floating cells were removed to obtain a monocyte-enriched culture that was treated with macrophage-colony stimulating factor. Seven days later, the cultures were infected with CV-B4 E2 or CV-B4 JVB (multiplicity of infection: 1); (**a**) 24 and 48 h post-infection, the cellular viability was measured using the Orangu^®^ viability assay, and results are expressed as the percentage of viability compared with controls (Mean ± SD, n = 13). (**b**) The supernatants are harvested 6 and 48 h post-infection to evaluate the level of infectious particles by titration, and the results are expressed as TCID50/mL; mean ± SD of log-transformed values are shown (*n* = 13). The dotted line indicates the threshold of the viral titration method (log 10^1.5^ TCID50/mL).

**Figure 6:**
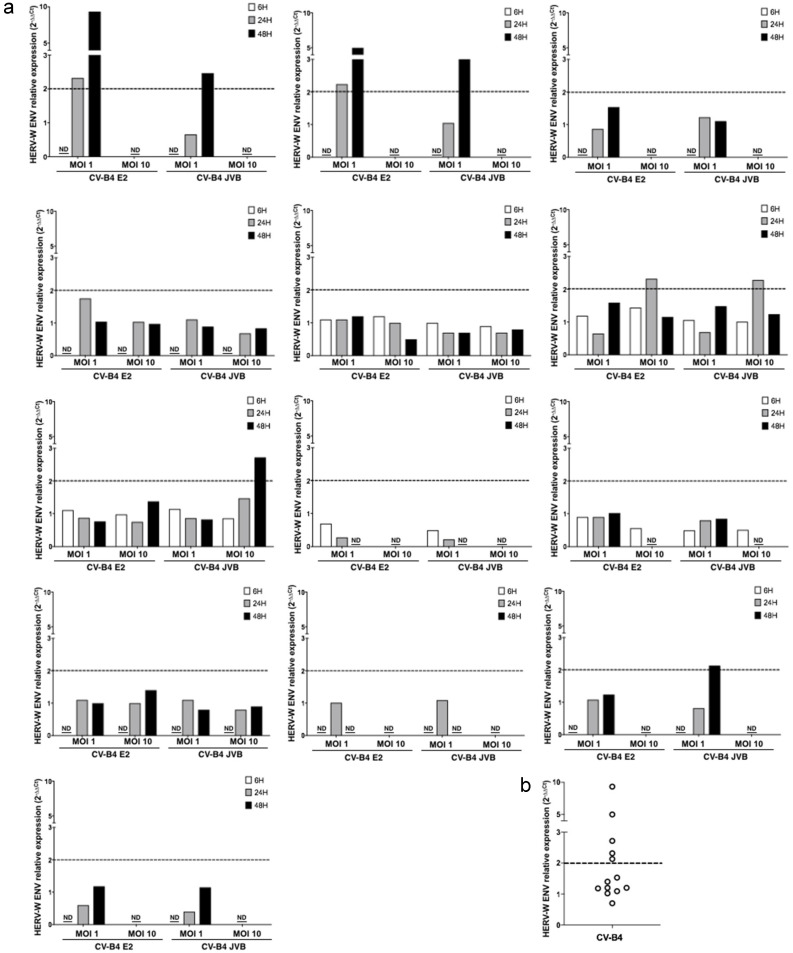
Individual representation of Human Endogenous Retrovirus W Envelope (HERV-W *ENV*) mRNA expression in macrophages infected by Coxsackievirus B4 (CV-B4). Macrophages from 13 healthy donors were prepared as previously described in the legend of Figure 5. Total RNA was extracted from the cells 6, 24, and 48 h post infection with TriReagent, and the level of intracellular HERV-W *ENV* mRNA in each culture was assessed in duplicate using RT-qPCR normalized with beta-actin mRNA expression. Each result is the mean of two or three determinations; furthermore, each RT-PCR datum is the mean of two technical duplicates. The relative expression of HERV-W *ENV* mRNA in CV-B4 E2-infected cultures compared with that in mock-infected cultures is calculated using the 2^-ΔΔCt^ method. (**a**) The individual relative expression of HERV-W *ENV* for each culture is shown. ND: not done due to an insufficient number of cells. (**b**) The maximum individual relative expression of HERV-W *ENV* for each culture is shown. The dashed line represents a relative expression of 2.

PBMCs were isolated from 13 buffy coats as described in Materials and Methods. The culture was rinsed to get rid of other cell types and obtain monocyte-enriched PBMCs as they attached to the surface of the plate; they were then treated with M-CSF to further induce complete differentiation of monocytes into macrophages. Macrophage cultures were then inoculated with CV-B4 E2 or CV-B4 JVB (MOI = 1 or MOI = 10) and incubated for 6, 24, and 48 h. Cell viability was assessed with Orangu^®^ as previously described. The viability of mock-infected and infected cell cultures was similar (Figure 5a). The infectious titers in the supernatants of cultures were 10^1.5^ and > 10^3^ TCID50/mL at 6 and 48 h post-infection, respectively (Figure 5b). In cultures of macrophages isolated from five of 13 buffy coats, the relative expression of HERV-W *ENV* in infected cultures was >2-fold either 24 or 48 h post-infection when inoculated with CV-B4 E2 or CV-B4 JVB at MOI = 1 or MOI = 10 (Figure 6).

## 4. Discussion

The effect of CV-B4 infection on HERV-W *ENV* expression in pancreatic ductal cells, PBMCs, and macrophages was investigated, with several noteworthy considerations. The exocrine fraction of pancreatic cells obtained from the pancreas of five brain-dead patients was cultivated in geneticin-complemented medium to limit fibroblast overgrowth. In vitro de/transdifferentiation of exocrine cells into ductal cells occurred as previously described by our team [30]. The infection of PBMCs with CV-B4 can be enhanced when the virus is mixed with diluted immune serum [24,25,34]. Indeed, in the present study, a higher yield of intracellular enteroviral RNA was obtained from PBMCs inoculated with CV-B4 mixed with immune serum diluted 1000-fold. The level of viral RNA in the monocyte-enriched cell population was determined using RT-qPCR. Monocytes were recovered after enrichment on the basis of their adherence to the plastic culture plate followed by successive rinsing steps to remove other cell types, which is a simple method to increase the proportion of monocytes as previously described [35,36].

Macrophages infected with CV-B4 produced viral particles as shown by the increase in the levels of infectious particles in the supernatants of cultures harvested 6 and 48 h post-infection; this finding agrees with that in previously reported studies [26]. Moreover, the intracellular enteroviral RNA was also detected using RT-qPCR in macrophages infected with CV-B4 E2 (data not shown).

The expression of HERV-W *ENV* was evaluated using RT-qPCR and normalized with a control housekeeping gene, as described previously [3]. The migration of PCR products on agarose gel as well as the dissociation curves of the amplicons made it possible to verify the specificity of the method (data not shown).

Compared with mock-infected PBMCs, an elevated expression of HERV-W *ENV* mRNA was observed when PBMCs of one donor were inoculated with CV-B4 E2 and when PBMCs of another were infected with the virus mixed with immune serum diluted 1000-fold. Immune serum enhanced the infection of PBMCs from three donors with CV-B4 E2 (relative expression >3); however, it resulted in an increase of HERV-W *ENV* in the cells from one donor only. Thus, the relative expression of HERV-W *ENV* was higher when the infection of cells was enhanced by the immune serum. Taken together, these observations suggest that CV-B4 E2 can induce the expression of HERV-W *ENV* in PBMCs and the immune serum can enhance the effect of CV-B4 E2 in total PBMC cultures.

Total RNA from infected macrophages was extracted 6, 24, and 48 h post-infection. The relative expression of the HERV-W *ENV* gene in infected macrophages compared with that in control macrophages was expressed by normalization with beta-actin mRNA. An increase in the expression of the HERV-W *ENV* gene with a ratio > 2 was obtained in the macrophage cultures of five donors when they were infected with CV-B4 (mean ± SD: 4.30 ± 3.03). The macrophage and pancreatic cell cultures were incubated in the presence of a HEp-2 cell lysate to check that the cellular proteins present in the viral suspensions derived from HEp-2 cell cultures did not induce the expression of HERV-W. The expression of the HERV-W *ENV* gene was not different in cells 6 h after infection compared with that observed in the corresponding control. In contrast, the expression of the gene was increased in cells 24 or 48 h post-infection, with an MOI of 1 or 10 in case of macrophages and with an MOI of 0.01, 0.1, or 1 in case of pancreatic cells. The hyperexpression of HERV-W ENV was induced by CV-B4 E2 in the cultures of macrophages and pancreatic ductal cells. CV-B4 JVB also caused this effect in cultures of macrophages, but it has not been tested in cultures of pancreatic cells.

CV-B4 infection stimulated the transcription of the HERV-W *ENV* gene in macrophage cultures of 5 out of 13 donors and in pancreatic cell cultures of 3 out of 5 donors. Interindividual variability in the expression of the HERV-W *ENV* gene has been observed. One cannot rule out the fact that the effect of CV-B4 infection on the expression of HERV-W ENV depends on genetic factors specific to each donor. Variability in the expression of the HERV-W *ENV* gene due to genetic factors has been observed in patients with MS [37]. In the human genome, owing to the variation of insertions and deletions during the evolutionary process, many copies of the HERV-W *ENV* gene are distributed over several chromosomes and in different loci, with non-ubiquitous copies that are not present in the genome of all humans [5]. The interindividual variability that we observed may be due to differences in response to CV-B4 infection or absence of copies of the HERV-W *ENV* gene that respond to this stimulation. These hypotheses should be explored to better comprehend the mechanisms of expression of the HERV-W *ENV* gene and the interindividual variability of response to a CV-B4 infection.

The level of HERV-W ENV protein in macrophages and pancreatic cells has been investigated using a very sensitive quantitative immunoblot technique allowing the detection of small quantities of proteins [3]. In pancreatic cells infected with CV-B4, the ENV protein was detected in a larger quantity than in the controls. In contrast, the amounts of protein in macrophages were not different, both in infected cultures and controls. However, the level of HERV-W *ENV* mRNA in infected macrophage cultures was higher than that detected in the controls, although we have not excluded the fact that the amount of ENV protein in infected macrophages was lower than the detection threshold of our method. The fold increase of ENV protein in infected pancreatic cells was significantly greater than the fold increase in the levels of HERV-W *ENV* in these cells, consistent with active translation and protein production. Furthermore, there was no correlation between increase in the ENV protein and increase in HERV-W *ENV* in macrophages. These observations suggest that CV-B4 infection has an impact not only on the transcription of HERV-W *ENV* but also possibly on the post-transcriptional, translational, and/or post-translational mechanisms. Depending on the modulation of these processes, the ENV protein can accumulate in cells or render itself undetectable.

An increased expression of the HERV-W *ENV* gene stimulated by DNA viruses has already been obtained in various in vitro systems: infection of astrocytic cells by Epstein Barr virus (EBV) [38] and infection of PBMCs by human herpesvirus-6 [11]. In addition, an increased expression of HERV-W ENV has been reported in PBMCs of individuals infected with EBV [39]. The influenza A virus has been shown to increase the expression of HERV genes, in particular, the *ENV* gene of nerve cells (SK-N-MC line) [40,41]. These DNA viruses and the influenza virus, which is an RNA virus, multiply in the nucleus of cells. CV-B4, in contrast, is a cytoplasmic RNA virus; the mechanisms involved in the effect of viral infections on the expression of HERV-W ENV should be explored. In preliminary experiments, it was observed that infectious CV-B4 was needed to increase HERV-W *ENV* mRNA. Nevertheless, it was reported in previous studies that infection with HHV-6 was not needed to activate HERV-W *ENV*, whereas the infection with HSV-1 was needed, at least the expression of IE genes [11,42].

In patients with T1D, the activation of HERV-W ENV has been demonstrated: the *ENV* mRNA level was higher than that in the PBMCs of control subjects, and the ENV protein was detected in the serum and exocrine pancreas of patients with T1D [3]. Furthermore, the activation of pol genes of HERV-W in PBMCs of patients with new-onset T1D was recently reported [43]. The HERV-W *ENV* genomic copy encoding the pathogenic HERV-W ENV protein involved in T1D remains to be identified. Interestingly, our results show that CVB4 infection can cause the activation of HERV-W ENV in human primary cells, including macrophages and pancreatic cells. These observations open up new avenues of reflection to investigate the viral pathogenesis of T1D. Infection with a virus, such as CV-B4, can result in the activation of an endogenous factor, in this case HERV-W ENV, whose pathogenic effects have been reported: inhibition of insulin secretion by β cells, induction of autoimmunity by molecular mimicry, and superantigen-like activity exacerbating the immune response against pancreatic cells [3,44,45,46]. The identification of particular HERV-W-*ENV* genomic copy activated by CV-B4 and a better understanding of the mechanisms of activation of HERV-W ENV by CV-B4 infection and especially of the virus and host factors involved in this interaction are warranted. Future studies will be directed along this line in our laboratory.

## Figures and Tables

**Table 1:** Oligonucleotide Primers for Human Endogenous Retrovirus W Envelope RT-PCR.

Oligonucleotide Primers	Sequence (5′–3′)	Number of Bases
HERV-W ENV_Forward	GTATGTCTGATGGGGGTGGAG	21
HERV-W ENV_Reverse	CTAGTCCTTTGTAGGGGCTAGAG	23
beta-actin_Forward	TTGCCGACAGGATGCAGAA	19
beta-actin_Reverse	GCCGATCCACACGGAGTACT	20

**Table 2:** Oligonucleotide Primers and Probe for Enteroviral RNA RT-qPCR.

Oligonucleotide Primers and Probe	Sequence (5′–3′)	Number of Bases
ENT_Forward	CCCTGAATGCGGCTAATC	18
ENT_Reverse	ATTGTCACCATAAGCAGC	18
Probe	AACCGACTACTTTGGGTGTCCGTGTTT	27
